# Natural language processing and network analysis in patients withdrawing from life-sustaining treatments: a retrospective cohort study

**DOI:** 10.1186/s12904-022-01119-8

**Published:** 2022-12-22

**Authors:** Wei-Chin Tsai, Yun-Cheng Tsai, Kuang-Cheng Kuo, Shao-Yi Cheng, Jaw-Shiun Tsai, Tai-Yuan Chiu, Hsien-Liang Huang

**Affiliations:** 1 Department of Family Medicine, National Taiwan University Hospital Hsin-Chu Branch, No. 25, Ln. 442, Sec. 1, Jingguo Rd., North Dist., Hsinchu City, 300 Taiwan (R.O.C.); 2 Department of Technology Application and Human Resource Development, National Taiwan Normal University, 162, Section 1, Heping E. Rd., Taipei City, 106 Taiwan (R.O.C.); 3 Department of Medicine, National Taiwan University, No.1 Jen Ai Road Section 1, Taipei, 100 Taiwan (R.O.C.); 4 Department of Family Medicine, College of Medicine and Hospital, National Taiwan University, 7 Chung-Shan South Road, Taipei, 100 Taiwan (R.O.C.)

**Keywords:** Withdrawing treatment, Palliative care, Natural language processing, Terminal care, Hospice care

## Abstract

**Background:**

Providing palliative care to patients who withdraw from life-sustaining treatments is crucial; however, delays or the absence of such services are prevalent. This study used natural language processing and network analysis to identify the role of medications as early palliative care referral triggers.

**Methods:**

We conducted a retrospective observational study of 119 adult patients receiving specialized palliative care after endotracheal tube withdrawal in intensive care units of a Taiwan-based medical center between July 2016 and June 2018. Patients were categorized into early integration and late referral groups based on the median survival time. Using natural language processing, we analyzed free texts from electronic health records. The Palliative trigger index was also calculated for comparison, and network analysis was performed to determine the co-occurrence of terms between the two groups.

**Results:**

Broad-spectrum antibiotics, antifungal agents, diuretics, and opioids had high Palliative trigger index. The most common co-occurrences in the early integration group were micafungin and voriconazole (co-correlation = 0.75). However, in the late referral group, piperacillin and penicillin were the most common co-occurrences (co-correlation = 0.843).

**Conclusion:**

Treatments for severe infections, chronic illnesses, and analgesics are possible triggers for specialized palliative care consultations. The Palliative trigger index and network analysis indicated the need for palliative care in patients withdrawing from life-sustaining treatments. This study recommends establishing a therapeutic control system based on computerized order entry and integrating it into a shared-decision model.

## Background

Life-sustaining treatments, including cardiopulmonary resuscitation, mechanical ventilation, and hemodialysis, are frequently employed in hospital intensive care units (ICUs). However, when patient deaths are inevitable, these treatments cannot reverse the underlying medical conditions and may even cause further harm [1]. Withdrawing life-sustaining treatments should be considered an option when healing or improving health is no longer possible. However, life-sustaining treatment withdrawal involves several considerations, including preparing for the withdrawal of life-sustaining measures, distress assessment and management, ethical and legal issues, and bereavement support [2]. It is a complex scenario not only for patients and their families but also for primary healthcare teams.

The integration of palliative care with critically ill patients before withdrawing life-sustaining treatment is a beneficial healthcare measure. First, palliative care consultants can help improve the quality and quantity of communication, facilitate shared decision-making (SDM), and provide goal-concordant care. One example is the SOP model (shared decision-making with oncologists and palliative care specialists), which significantly increases the documentation rate of Do Not Resuscitate Order (DNR) preferences in patients with advanced cancer [3]. Second, palliative care interventions can help decrease symptoms of distress and anxiety, thereby providing high-quality end-of-life care without affecting hospital mortality [4–6]. Despite these benefits, delays or the absence of palliative care are still common [7].

It has been challenging for clinicians to determine the appropriate timing for patient consultation with palliative specialists. Prognostic uncertainty, fear of causing distress, navigating patient readiness, and feeling unprepared for conversations are all possible barriers [8]. To identify patients who are appropriate for palliative consultation, previous studies have used screening criteria (also known as “triggers”) [9]. In a multi-center, multidisciplinary survey of critical care clinician attitudes, the acceptable triggers were metastatic malignancy, unrealistic goals of care, or persistent organ failure [7]. This study employed natural language processing and network analysis as novel methods to determine the triggers of palliative care.

Natural language processing (NLP) refers to computational methods that enable machines to process and analyze written texts. Through NLP, unstructured free-text medical notes can be rapidly scanned to detect prespecified indicators. NLP has been applied in several areas of medical research. For example, it was applied to the analysis of suicidal ideation and attempts, classification of incident reports, and adverse events in healthcare [10, 11]. A study also showed that NLP successfully identified gastrostomy indications with an accuracy level similar to human coders [12]. Network analysis can help identify various association patterns and visualize the relationships of a dataset in one graph [13]. In an observational study, network analysis of palliative care patient-reported outcome measure (PROMs) data provided functional information to support timely decision-making [14].

This study aimed to identify triggers associated with early palliative team consultation to help clinicians determine the appropriate timing to initiate palliative care. We used NLP and network analysis to analyze the medical records of patients who withdrew from life-sustaining measures. Unlike previous studies on palliative care triggers that focused on patient characteristics and diagnoses, this study focused on identifying medications as palliative triggers. Once triggers are identified, they can be integrated into an SDM model and applied to therapeutic control systems in the future.

## Methods

This retrospective analysis of patients who underwent endotracheal tube withdrawal and were under specialized palliative care in the ICUs of a medical center in Taiwan was conducted between July 2016 and June 2018. We identified the date of the patient’s first referral to the palliative team and calculated the interval between their first referral and death. Since the data were skewed to the right, we separated all patients based on the median survival time into the early integration group (≥ 22 days) and the late referral group (< 22 days).

We used text mining tools and applied NLP methods in R (Computer vision Principles, Algorithms, Applications, Learning Book, 5th Edition, 2018) to analyze the medical records of patients from the admission date to the expiration date. The medical records included admission notes, weekly summaries, free notes, and hospice notes. We selected the drug names as our “words of interest.” We retrieved all drug names from the “Drug Index A to Z” on Drugs.com (https://www.drugs.com/drug_information.html) by web scraping using R(3.6.0.). The index lists over 24,000 prescriptions, including generic and brand names. One palliative specialist and one family medicine specialist received palliative training combined with synonyms (e.g., fluconazole and Diflucan; cefepime and Maxipime). A document-term matrix was generated for each patient. Subsequently, we used NLP to compare the frequency of “words of interest” between the early integration and late referral groups.

### Natural language processing

Our NLP pipeline is a text-mining component that performs a particular language analysis that helps machines to read. Unstructured text data probably takes a lot of time and resources. Material cleaning must be processed first, especially in languages made up of orthographies, such as English, punctuation, spaces, tenses, singular, and plural. Unlike structured data, unstructured data have no static fields, and we make the pipeline suitable for determining the relationships between features. We use analysis models for the text mining groups “tm,” “co-occur,” and “dplyr.” Further, we extracted keywords from the data and created a co-occurrence matrix. Co-occurrence is a square matrix that describes the co-occurrence of two terms in context. Therefore, co-occurrence matrices are sometimes called term matrices and are square matrices because they are matrices between each term and the other. The disadvantage of the word context matrix is that it does not consider comments that are similar but in different sentences.

**Fig. 1:**
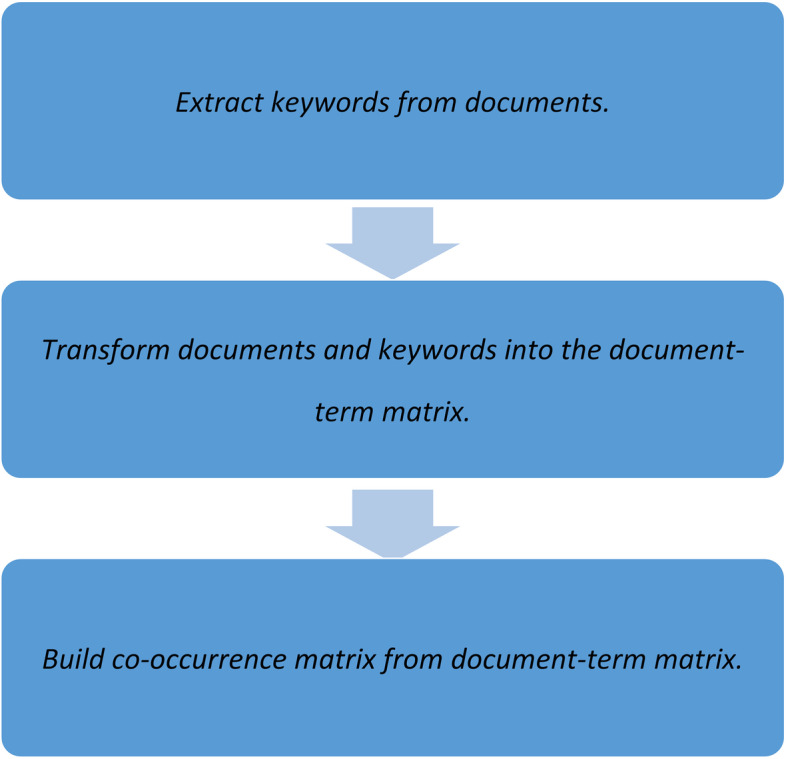
The steps of natural language processing. This figure explains the steps of natural language processing. We turn to keyword extraction to find essential information from a specific document. When these keywords came from different documents, we converted the documents and corresponding keywords into a document-term matrix. The co-occurrence matrix follows the equation. C = A’A, where C is the co-occurrence matrix, A is the document-term matrix, A’ is the transpose of the A matrix, and A’A is the matrix multiplication of A’ and A. They contain a count of the times a given feature occurs in the documents’ relationship with another given feature

The steps of NLP are shown in Fig. 1.

- Step 1. Extracting keywords from documents: We usually turn to keyword extraction when we want to find essential information from a specific document.
- Step 2. Transform documents and keywords into a document-term matrix: We can extract keywords from a single document into multiple documents. When these keywords came from different documents, we converted the documents and corresponding keywords into a document-term matrix. The matrix entry is the number of times a keyword appears in its documents. For example:

**Table:** 

	albumin	vancomycin	fentanyl	furosemide	…
Document1	1	1	1	1	
Document2	1	0	0	1	
Document3	1	1	0	0	
…

- Step 3. Build a co-occurrence matrix from the document-term matrix. The co-occurrence matrix follows the equation. C = A’A, where C is the co-occurrence matrix, A is the document-term matrix, A’ is the transpose of the A matrix, and A’A is the matrix multiplication of A’ and A. They contain a count of the times a given feature occurs in the documents’ relationship with another given feature.

### Palliative trigger index

The universal use of medication in the early integration group but not in the late referral group reflects the potential of being an early palliative consultation trigger. Moreover, the higher the word frequency in the early integration group, the more representative it was. Thus, we created the Palliative trigger index for the purpose of natural language processing and network analysis in our study based on a literature review, authors’ clinical experience, and expert opinions, which included experts from medicine, big data management, and technology application. This index is the product of word frequency in the early integration group and the difference in word frequency between the early integration and late referral groups.

The formula is as follows:

$$\mathrm{Palliative}\;\mathrm{trigger}\;\mathrm{index}\:=\:\mathrm{Word}\;\mathrm{frequency}\;\mathrm{of}\;\mathrm{early}\;\mathrm{integration}\;\mathrm{group}\;\ast\;\mathrm{Difference}$$

Words with a higher Palliative trigger index were considered better palliative triggers. If the difference or early group word frequency is zero, then the Palliative trigger index is zero, which is the minimum value.

### Network analysis

Network analysis made co-occurrence matrix visualization so that we could easily identify words that often appear together in the same patient’s medical records. The size of each dot indicates the frequency of a medical term. The lines indicate at least moderate co-occurrence (correlation ≥ 0.5); the stronger the co-occurrence, the thicker the line.

### Shared-decision-making model

**Fig. 2:**
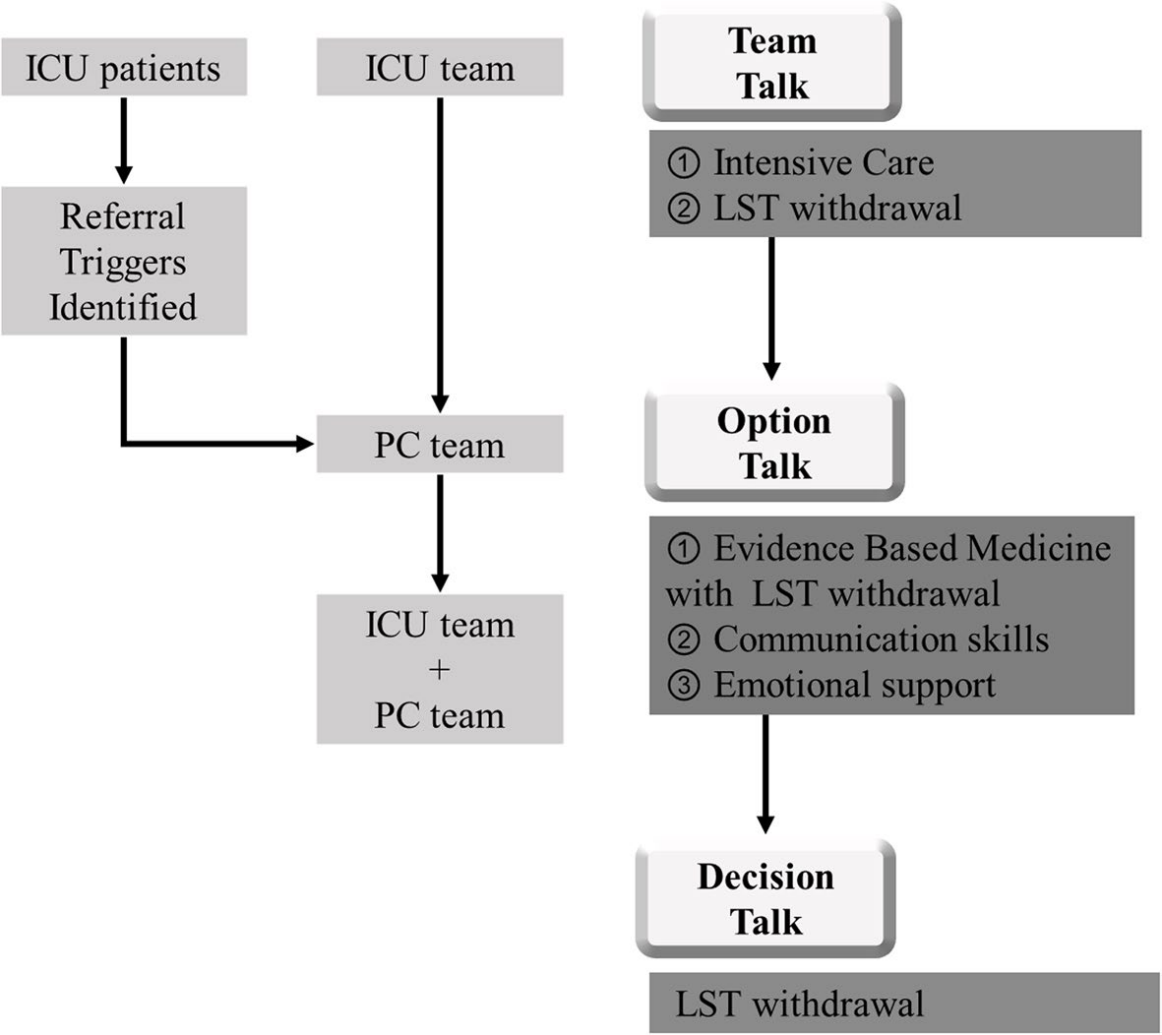
Framework of Intensive Care Unit (ICU) patients’ shared decision-making model on withdrawal of life-sustaining treatments. The SDM model for the patients was conceptualized using the three-talk model, including “team talk,” “option talk,” and “decision talk.” First, the ICU team would conduct “team talks” on ICU patients and their surrogates. After the ICU team identified the referral triggers of the patients, the patients or surrogates received the “option talk” from multidisciplinary teams with components of evidence-based medicine, communication skills, and emotional support to help reach a preference-based decision to withdraw life-sustaining treatments. After the last step, “decision talk,” the final decision is made. PC team = Palliative care team. LST = Life-sustaining treatments

A shared-decision-making model, with the cooperation of the ICU team and palliative care team, was implemented in our hospital, as shown in Fig. 2, to help early integration of palliative care for patients withdrawing from life-sustaining treatments. The model is based on a previous design for advanced cancer patients [3, 15, 16] and was modified for non-cancer patients by the authors of this study. The SDM for the patients was conceptualized using the three-talk model, including “team talk,” “option talk,” and “decision talk” [17, 18]. First, the ICU team would conduct team talks” for ICU patients and their surrogates. Once the referral of triggers of the patients were identified by the ICU team, the patients or surrogates would receive the “option talk” from the multidisciplinary team. They included components of evidence-based medicine, communication skills, and emotional support to help reach a preference-based decision on withdrawing life-sustaining treatments. Ensuing the last step, “decision talk,” the final decision is made.

## Results

### Study sample and baseline characteristics

**Fig. 3:**
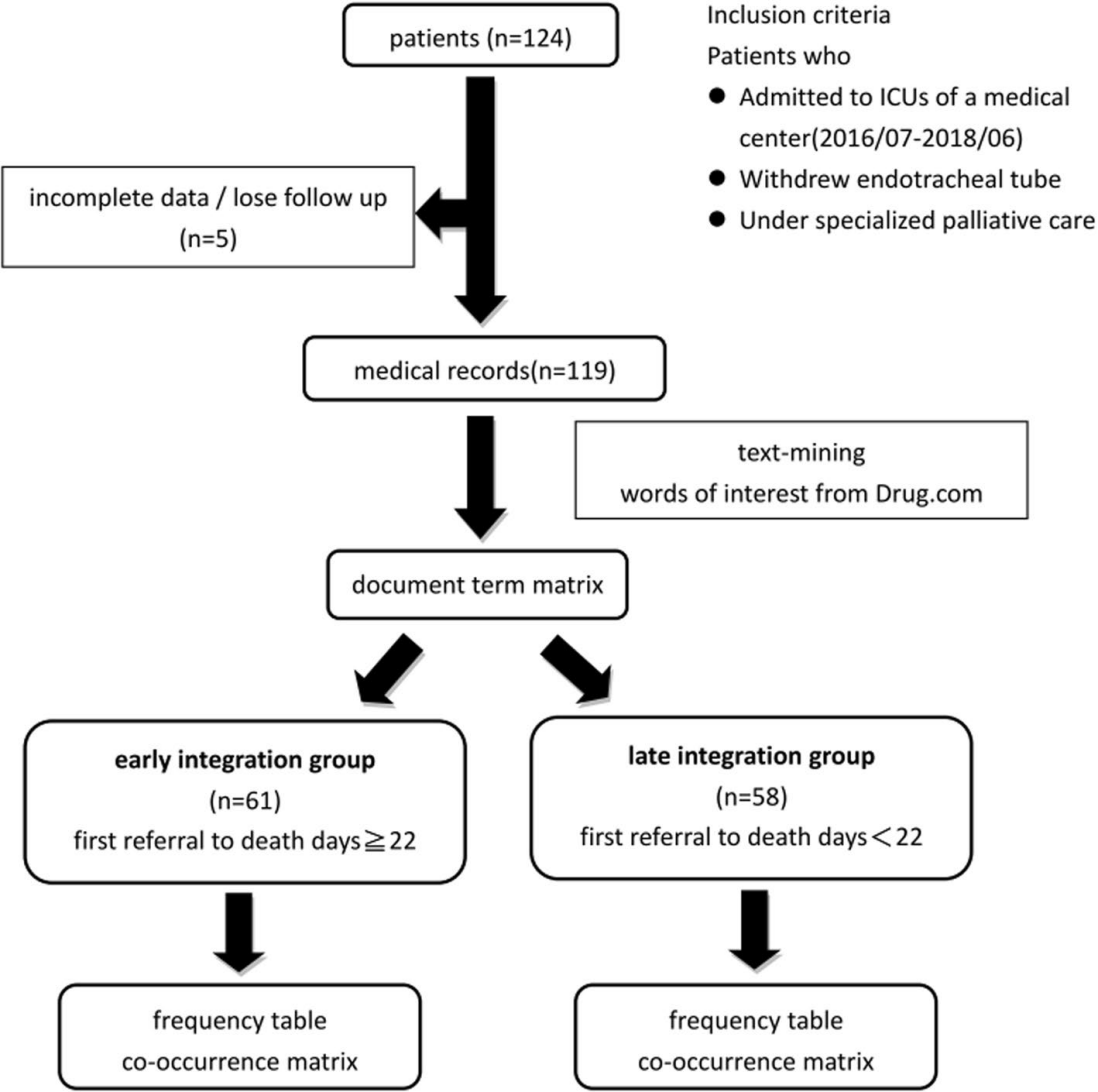
Research workflow. We used text mining tools and applied natural language processing to analyze medical records. A document-term matrix was generated for each patient. We separated all patients by the median survival rate (first referral to death) into an early integration group (≥ 22 days) and a late referral group (< 22 days). Based on the grouping results, we compared the word frequency and co-occurrence matrices

A patient recruitment flowchart is shown in Fig. 3. We identified 124 patients who met the study criteria. After excluding patients with incomplete medical records or those lost to follow-up, 119 were included in the final analysis. A total of 61 patients were categorized into the early integration group (≥ 22 days), and the remaining 58 patients were categorized into the late referral group (< 22 days).

The mean age of the entire cohort was 66 years (SD = 15.8), and more than half of the patients were men (60.5%; *n* = 72). Of the patients, 55.5% (*n* = 66) had hypertension and 46.2% (*n* = 55) had cardiovascular disease. Approximately 44.5% of patients were diagnosed with cancer (*n* = 53). The most common types of cancer on the list were lung (11.8%, *n* = 14), gastrointestinal tract (7.6%, *n* = 9), and head and neck (7.6%, *n* = 9) cancers. Most patients (69.7%; *n* = 83) received palliative care no more than a month before death.

### Palliative trigger index

Based on the study results, the top ten medications with a high Palliative trigger index are shown in Table 1. They included antibiotics such as cefepime, vancomycin, ceftazidime, antifungal agents, opioids, furosemide, albumin, and amiodarone. The rank of word frequency in the early integration group and the differences between the two groups are also included.

**Table 1:** Word frequency table and the Palliative trigger index (PTI)

	Early integration group	Group Rank	Difference	Difference Rank	Palliative Trigger Index	PTI Rank
Cefepime	35	1	16	4	560	1
Fluconazole	25	5	17	1	425	2
Vancomycin	22	9	17	1	374	3
Ceftazidime	21	10	17	1	357	4
Furosemide	23	6	15	5	345	5
Morphine	29	4	10	13	290	6
Albumin	23	6	12	8	276	7
Amiodarone	23	6	11	11	253	8
Fentanyl	19	11	11	11	209	9
Meropenem	16	15	12	8	192	10
Metronidazole	16	15	12	8	192	10

Palliative trigger index = Early integration group word frequency x Difference

Difference = Early integration group word frequency – Late referral group word frequency

### Network analysis

**Fig. 4:**
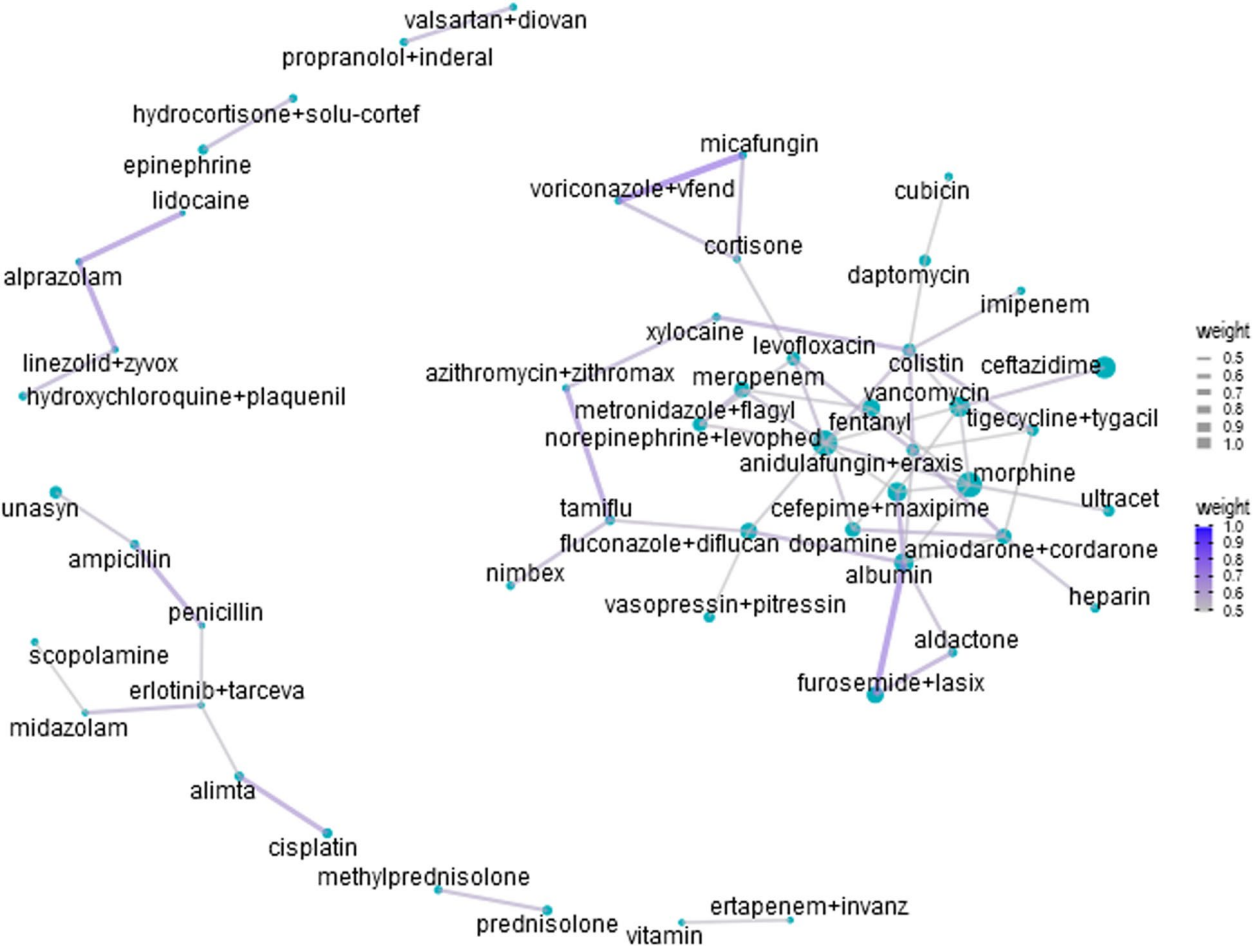
Network analysis of drugs of the early integration group. The size of each dot indicates the frequency of each medical term. The lines indicate at least a moderate co-occurrence (co-correlation ≥ 0.5). For visualization, if the co-occurrence was stronger, the line was thicker, and the blue color was deeper

Figure 4 shows the network analysis of the medications in the early integration group. Words with a frequency of fewer than three times were excluded. The top four co-occurrences in the early integration group were micafungin and voriconazole (co-correlation = 0.75), furosemide and albumin (co-correlation = 0.718), lidocaine and alprazolam (co-correlation = 0.667), and linezolid and alprazolam (co-correlation = 0.667).

**Fig. 5:**
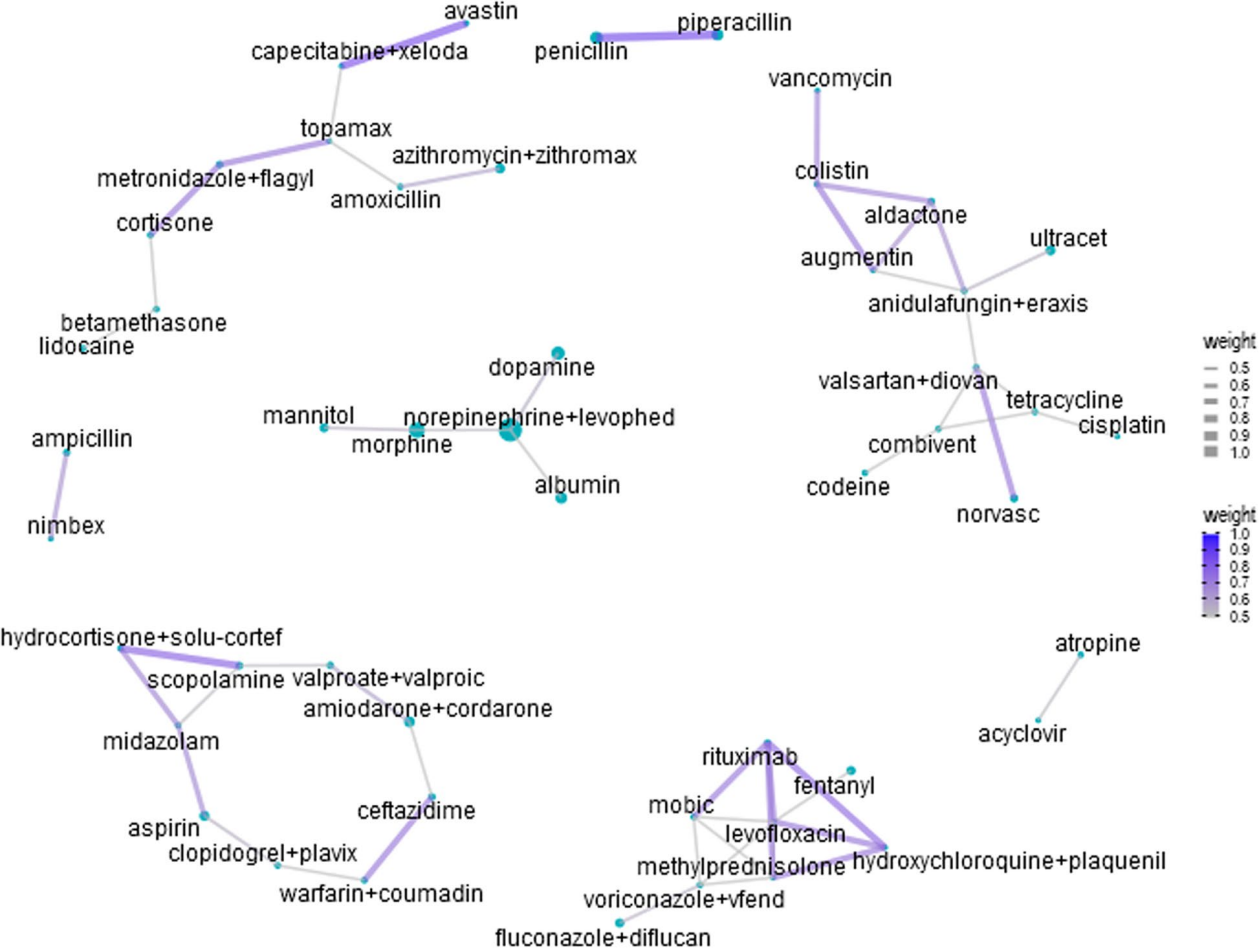
Network analysis of drugs of the late referral group. The size of each dot indicates the frequency of each medical term. The lines indicate at least a moderate co-occurrence (co-correlation ≥ 0.5). For visualization, if the co-occurrence was stronger, the line was drawn thicker and the blue color deeper

Figure 5 shows the network analysis of the medications in the late referral group. Words with a frequency of fewer than two times were removed. The top three co-occurrences in the late referral group were piperacillin and penicillin (co-correlation = 0.843); bevacizumab and capecitabine (co-correlation = 0.816); scopolamine and hydrocortisone (co-correlation = 0.775); and hydroxychloroquine and rituximab (co-correlation = 0.75).

## Discussion

The present study employed novel methods, including NLP and network analysis, to help physicians provide timely palliative care for patients withdrawing from life-sustaining treatments. The study results suggest that broad-spectrum antibiotics, antifungal agents, diuretics, albumin, and opioids are associated with early palliative consultation. Patients and families who withdraw life-sustaining treatments experience physical, emotional, and psychological challenges. Proper and timely palliative care referrals can help establish treatment goals, improve symptom control, and provide psychological support to patients and their families. However, in current clinical practice, most patients receive palliative care extremely late in the end-of-life stage.

Although early palliative care has gradually gained acceptance, the widespread integration of palliative care with standard medical treatment remains insufficient [19]. Therefore, the early recognition of patients’ palliative needs and possible triggers is important for achieving positive healthcare outcomes. Previous studies have reported that palliative triggers, such as advanced or life-limiting diseases, as well as characteristics of hospitalization, are associated with higher resource utilization and negative outcomes [20]. The medication-related palliative triggers identified by the NLP and network analysis in this study can be applied to computer triage systems in the future.

Medications with a high Palliative trigger index included antibiotics (cefepime, vancomycin, and ceftazidime), antifungal agents (fluconazole), opioids (morphine and fentanyl), furosemide, albumin, and amiodarone. The use of broad-spectrum antibiotics and antifungal agents indicated that the patient had a severe infection. Severe sepsis is the leading cause of death in ICUs [21]. Opioids are the primary treatment for pain related to advanced and progressive diseases, especially when withdrawing life-sustaining measures [22]. Furosemide and albumin are used to treat fluid retention [23]. The possible etiologies of fluid retention included fluid infusions during acute resuscitation, chronic cardiac disease, and renal disease, among others [23, 24]. Amiodarone is a potent antiarrhythmic agent used to treat ventricular arrhythmias and atrial fibrillation [25]. Overall, the use of medications with a high Palliative trigger index indicated that the patient was critically ill. Therefore, these data can serve as a reasonable trigger for physicians to recommend timely palliative care.

Network analysis enables the categorization of medications that often occur together in the medical records of the same patient. The co-occurrence of micafungin and voriconazole in the early integration group could be explained by the synergistic effects of severe systemic fungal infections in severely ill patients. Clinicians should adjust antifungal agents according to cultural evidence and clinical presentation, thereby increasing the co-occurrence of various antifungal agents. A possible explanation for the co-occurrence of albumin and furosemide in the early integration group is that both drugs are usually co-administered to correct the reduced oncotic pressure and reinforce their therapeutic effect [26].

In the late referral group, piperacillin and penicillin showed high co-occurrence. Piperacillin-tazobactam is a common choice for both directed and empirical treatment of critically ill patients [27]. Piperacillin is a broad-spectrum penicillin antibiotic, which may explain its co-occurrence. Bevacizumab and capecitabine also showed a high co-occurrence in the late referral group. The combination of bevacizumab and capecitabine is an effective and well-tolerated regimen for elderly patients with metastatic colorectal cancer [28]. This co-occurrence indicates that the patients were undergoing aggressive anticancer treatments.

Network analysis revealed several important patterns of medication use. Interestingly, there was some overlap between the high co-occurrence medications in the early integration group and high Palliative trigger index medications, such as albumin, furosemide, and antifungal agents. This observation reveals that high Palliative trigger index medications are often prescribed together, which indicates that these drugs share some common characteristics.

NLP can be used to automatically extract clinically relevant information, thereby reducing the time required by clinicians to extract salient information manually. In the future, these medical term data can be used to establish a triage system to identify the initial priorities of clinicians for the early integration of specialist palliative care. According to a previous study, a computerized order-entry-based therapeutic control system can remind physicians to perform Hepatitis B virus (HBV) screening before prescribing chemotherapy [29]. We may also apply a similar computerized order-entry-based therapeutic control system to integrate palliative care before the patient withdraws from life-sustaining treatments. When physicians prescribe broad-spectrum antibiotics, antifungal agents, or opioids, the computerized order-entry-based therapeutic control system can prompt them to evaluate whether the palliative team should be consulted. Furthermore, referral triggers can also be integrated into SDM. Palliative consultants and multidisciplinary teams can assist ICU teams in imparting high-quality communication, exploring all possible preferences, and making sound clinical decisions.

### Limitations

Although the present study reveals important findings, it has several limitations.

First, it was a single-center study. The sample size was relatively small, and only 119 patients were included in the final analysis. The participants only included Taiwanese citizens; therefore, the results may not reflect populations in other countries.

In addition, owing to the retrospective nature of the study, the causality between medications and palliative referral cannot be fully confirmed. The admission course of the early integration group might have been longer than that of the late referral group, which may have caused a possible bias because the early integration group had more opportunities for different medical terms in their medical notes.

In addition, there are many synonyms in use, including generic names, brand names, and abbreviations, which increases the difficulty of machine identification. Currently, synonyms, such as generic names and brand names, must be manually combined. The present study did not include patient symptoms or psychosocial or cultural factors in our analysis. As many of these factors were recorded in Chinese, they may have increased the difficulty of the analysis. The accuracy of NLP requires further improvement in future studies.

Patients with different diagnoses, in addition to medications, have different treatment courses and may influence end-of-life care. In this study, we did not perform disease-group-based NLP, which could be a potential source of bias. However, we believe that all patients in ICUs receiving life-sustained withdrawal treatments were in the terminal stages and had a similar prognosis and time for palliative care referral, regardless of disease type. In addition, the sample size for each disease was small, making it difficult to determine if a particular result is a true finding. Thus, we focused on all ICU patients who underwent endotracheal tube withdrawal and did not perform a subgroup analysis.

The Palliative trigger index is a novel method for evaluating palliative triggers. But it still needs further reliability and validity analysis in the future. High Palliative Trigger Index words could also be found in ICU patients who are not approaching to the end of life soon. Additional studies should be performed to check the incidence and value of the Palliative trigger index in non-end-of-life ICU patients to confirm our findings. Future studies can also extend the word of interest to NLP, enroll more participants, and focus on the outcomes of patients after the application of a therapeutic control system based on the computerized order entry.

## Conclusion

The use of NLP and network analysis is a novel method in critical health care that integrates palliative care research. NLP data are useful in identifying the characteristics of patients who withdraw life-sustaining treatments and the possible trigger factors that physicians must consider for palliative care referral. Treatments for severe infection, chronic illness, and analgesics are potential triggers for specialized palliative care consultations. Network analysis helps determine the relationships between each medication, enhancing the understanding of patients’ characteristics. The results of this study can help establish a therapeutic control system based on computerized order entry and integrate it into the shared-decision model, prompting proactive early palliative consultation. Furthermore, NLP and network analysis tools can be applied to effectively analyze large numbers of documents in various medical care fields.

## Data Availability

The datasets generated and analyzed during the current study are available from the corresponding author upon reasonable request.

## Abbreviations

NLP: Natural language processing
ICUs: Intensive care units
DNR: Do-not-resuscitate
